# Effect of popular songs from the reminiscence bump as autobiographical memory cues in aging: a preliminary study using EEG

**DOI:** 10.3389/fnins.2023.1300751

**Published:** 2024-01-09

**Authors:** Maria Cruz Martínez-Saez, Laura Ros, Marco López-Cano, Marta Nieto, Beatriz Navarro, Jose Miguel Latorre

**Affiliations:** ^1^Department of Psychology, Faculty of Medicine, University of Castilla La Mancha, Albacete, Spain; ^2^Applied Cognitive Psychology Laboratory, Research Institute for Neurological Disabilities, University of Castilla La Mancha, Albacete, Spain

**Keywords:** autobiographical memory, songs, electrical brain activity, EEG, spectral power, reminiscence bump, elderly

## Abstract

**Introduction:**

Music has the capacity to evoke emotions and memories. This capacity is influenced by whether or not the music is from the reminiscence bump (RB) period. However, research on the neural correlates of the processes of evoking autobiographical memories through songs is scant. The aim of this study was to analyze the differences at the level of frequency band activation in two situations: (1) whether or not the song is able to generate a memory; and (2) whether or not the song is from the RB period.

**Methods:**

A total of 35 older adults (22 women, age range: 61–73 years) listened to 10 thirty-second musical clips that coincided with the period of their RB and 10 from the immediately subsequent 5 years (non-RB). To record the EEG signal, a brain-computer interface (BCI) with 14 channels was used. The signal was recorded during the 30-seconds of listening to each music clip.

**Results:**

The results showed differences in the activation levels of the frequency bands in the frontal and temporal regions. It was also found that the non-retrieval of a memory in response to a song clip showed a greater activation of low frequency waves in the frontal region, compared to the trials that did generate a memory.

**Discussion:**

These results suggest the importance of analyzing not only brain activation, but also neuronal functional connectivity at older ages, in order to better understand cognitive and emotional functions in aging.

## 1 Introduction

Music clips have been shown to be an effective tool for eliciting autobiographical memories associated with emotional states in different types of populations (Schulkind and Woldorf, 2005; Janata et al., 2007; Ford et al., 2016). They have also been successfully used in older adults (López-Cano et al., 2020). Furthermore, popular music clips have been found to be useful for studying the organization and structure of autobiographical memories (Janata et al., 2007). However, little research has been conducted on the neural correlates of the induction processes of such memories, especially in the field of aging. Therefore, adopting a novel perspective, the present work focuses on analyzing the neural correlates of the process of induction of autobiographical memories through popular songs in a sample of older adults, using electroencephalography (EEG).

### 1.1 Autobiographical memory

Autobiographical memory (AM) is a type of declarative memory that refers to the recall of personal experiences from an individual’s past (Williams et al., 2007). It is a fundamental construct for human functioning that plays a role in developing a sense of self, problem solving, goal attainment, and personal well-being (Conway and Pleydell-Pearce, 2000; Waters, 2014).

Autobiographical memory has semantic and episodic aspects (e.g., Baddeley, 2012; Mace, 2019). The semantic component refers to general knowledge about the world (e.g., “Madrid is the capital of Spain”), being an abstract and conceptual domain that generates the retrieval of generic memories. In contrast, episodic memory involves the first-person re-experiencing of a specific event (e.g., “I went to the Prado Museum on my study trip to Madrid”). Episodic autobiographical memories contain contextualized details, including sensory-perceptual information, thoughts, images, and emotions.

The AM system is hierarchically structured into different levels of specificity (Conway and Pleydell-Pearce, 2000). At the top level are extended memories, which are general memories with a duration of more than 1 day (e.g., “The weekend I spent with my friends at the beach”) and categoric memories, which are general and recurrent memories grouped into categories (e.g., “Christmas dinners with my family”). The bottom level of the autobiographical hierarchy comprises specific memories, which are personally significant memories associated with a specific place and time, the duration of which is 24 h or less (e.g., “The day I met my partner”).

### 1.2 Autobiographical memory induction

Specific memories can be generated by means of two types of processes: direct retrieval and generative retrieval. In the former, the memory appears spontaneously in response to an environmental cue (e.g., smells, sounds), while in generative retrieval the memory emerges through a top-down search process. That is, the memory process gradually moves down from general memories to events involving specific knowledge. This second process demands more cognitive effort and time than direct retrieval, as it is based on a more complex methodology and is that most widely used in AM research (Griffith et al., 2012). More specifically, generative retrieval frequently makes use of cues, such as words, pictures, and music. Thus, listening to music can evoke memories associated with certain life periods, being known as music-evoked autobiographical memories (MEAMs; Janata et al., 2007; Cady et al., 2008; Janata, 2009; Ford et al., 2011). MEAMs are a special case of autobiographical memories, being self-defining memories related to a song. Subsequently, the song becomes a cue that triggers autobiographical retrieval (Janata et al., 2007).

Recently, several studies have implemented experiments designed to analyze the autobiographical salience of songs considering their release date, with an approach focused on the application of the reminiscence bump (RB) concept to musical memory, whereby older individuals disproportionately recall events from adolescence and adulthood (between 15 and 30 years of age) compared to other life stages. This is related to the large number of significant life events that typically occur during this period, such as leaving the childhood home, completing studies, getting one’s first job, and starting a family (Rubin et al., 1998). The results obtained have shown that the songs released in a participant’s RB period are the preferred ones, the most easily recognized and, therefore, the ones that trigger more MEAMs (Krumhansl, 2017; Rathbone et al., 2017; López-Cano et al., 2020). In this sense, the study by López-Cano et al. (2020), conducted with older adults (Experiment 2), found that around 70% of the songs from the RB period used in their work evoked some kind of autobiographical memory, while only 44% of the songs not from that period successfully evoked a memory.

### 1.3 Neural bases of autobiographical memory induction by musical stimuli

The multimodal nature of AM reflects the complexity of its neural substrates. Neuroimaging studies have consistently identified a set of regions that are coactivated during autobiographical recall, including the medial and lateral part of the temporal, frontal, and parietal cortex as well as limbic structures (e.g., Svoboda et al., 2006; Fossati, 2013; Lee et al., 2021). Importantly, the hippocampus coordinates the aspects that form retrieved autobiographical memories, determining how they are reconstructed in the mind (Sheldon et al., 2019) and dictating the level of detail and their personal meaning (Addis et al., 2004). Additionally, the network configured by the ventromedial prefrontal cortex (vmPFC), hippocampus and amygdala has been consistently associated with AM and affective processes (Buchanan, 2007; Nawa and Ando, 2019).

By analyzing the EEG signal, Schaefer et al. (2013) evidenced a component in the temporal cortex that appeared to be related to the onset of a perceived stimulus, and parietal and frontal-central components that showed an initial activation generated by the associated images. Additionally, Ding et al. (2019), analyzing the electrocortigraphic signal, showed the temporal sequence of the gamma band during music listening and recall. The response to listening to music is initiated by external auditory stimuli that first reach the sensory cortex (i.e., auditory cortex in the temporal region) and, subsequently, the frontal region. Information flowing from the sensory cortex to the frontal cortex is a bottom-up process. In contrast, music recall initializes responses starting from the frontal cortex and ending in the sensory cortex, reflecting a top-down process.

Regarding the induction of memories of an emotional character, the limited studies on this question suggest that when we listen to familiar songs from our personal past, the association between music and memories is located in the dorsolateral medial prefrontal cortex, which acts in conjunction with other areas of the prefrontal cortex in tonality tracking and in the response to familiar songs and the autobiographical memories they are associated with (Janata, 2009).

With respect to frequency bands, studies report that the beta band, particularly in its higher ranges, plays a key role in music processing (Petsche et al., 1993) and emotional arousal modulation (Aftanas et al., 2006; Hadjidimitriou and Hadjileontiadis, 2012). Meanwhile, alpha oscillations are prominent during AM retrieval (Manns et al., 2018), with the asymmetry of alpha band oscillations in the prefrontal cortex being the index for discrimination between positively and negatively valenced emotions (Schmidt and Trainor, 2001; Davidson, 2004). Finally, the theta power band reflects the affective processing generated during music listening (Sammler et al., 2007), while the gamma band is primarily related to arousal effects (Keil et al., 2001; Balconi and Lucchiari, 2008; Hadjidimitriou and Hadjileontiadis, 2012).

It has generally been reported when music is used as a tool to elicit autobiographical memories, participants retrieve memories of different levels of specificity, allowing us to differentiate the brain activity associated with each of them. Retrieval of events related to personal self-schemas occurs in the dorsolateral and dorsomedial prefrontal regions, while recall of specific memories is associated with greater activity in the bilateral medial temporal lobe and dorsomedial prefrontal region, compared to retrieval of general memories. These results suggest that initial memory search processes at different levels of the autobiographical memory hierarchy activate different components of this memory system (Ford et al., 2011).

As well as the studies on the role of music in generating autobiographical memories, reference must be made to those analyzing the neural processes related to the emotional valence of music. Specifically, studies using EEG methodology have suggested that listening to highly pleasant music is associated with (i) a relatively higher oscillatory activity in the theta band over the frontocentral area (FC) and in the alpha band over the parieto-occipital area; and (ii) with a progressive increase in oscillatory power over time (Nemati et al., 2019). In this sense, it seems that listening to pleasant music demands greater attention, which leads to a decrease in memory performance. The gradual development of low-frequency oscillations in the frontal and posterior regions may be due, at least partly, to the progressive recruitment of higher levels of attention in response to pleasurable music. These findings show that slow frontotemporal loops play a central role in the pleasantness evoked by music (Ara and Marco-Pallarés, 2020). Finally, it has also been suggested that right frontotemporal theta phase synchronization is positively associated with the pleasure induced by listening to unfamiliar music. In contrast, temporoparietal interhemispheric synchronization is positively associated when the pleasant music is familiar. These results shed light on the possible oscillatory mechanisms underlying frontotemporal and temporoparietal connectivity and their association with music-evoked pleasantness and familiarity (Ara and Marco-Pallarés, 2021).

In short, the neural bases of listening to songs associated with autobiographical memories have three components: (i) those involved in the recognition and familiarity of the music; (ii) those involved in accessing the autobiographical memories and the different levels of specificity; and (iii) those involved in the emotional valence induced by the music and/or the memory. These processes have primarily been studied using different EEG techniques (Medina et al., 2018). In this sense, it is worth noting that the analysis of EEG signals allows us to ascertain the most representative characteristics used to identify the different functions linked to brain activity. One of the most widely used techniques is Power Spectral Density (PSD), which provides information on the power distribution of a signal across its different frequency bands (Medina et al., 2018): delta (1 ≤ δ < 4 Hz), theta (4 ≤ θ < 8 Hz), alpha (8 ≤ α < 13 Hz), beta (13 ≤ β < 32 Hz) and gamma (32 ≤ γ < 45 Hz). The main advantage of this type of analysis is that it facilitates the study of the characteristics of each of the frequency bands separately as each band is related to a different brain activity (Sciotto and Niripil, 2014).

### 1.4 The current study

Drawing on the above ideas and considering the scarcity of this type of studies in older populations, the aim of this work is to analyze the patterns of brain electrical activation in older adults during a task involving listening to songs related to autobiographical memories. Previous studies (López-Cano et al., 2020) have shown that songs from the RB period elicit more memories than those from later stages of life (non-RB), although knowledge of what happens in these cases in terms of activation of frequency bands is still scant. Consequently, this study has two main objectives: (1) to determine whether there are differences in frequency bands in music clips that generate memories versus those that do not; and (2) to ascertain whether there are differences in frequency bands when listening to music clips from the RB period versus those from a later stage of life. Additionally, we also pretend to analyze the association between the power spectral bands and music clips as an autobiographical memory cue.

To answer these questions, this study intended to analyze the EEG signals collected in an experiment to induce emotions and autobiographical memories through popular songs, following the procedure proposed by López-Cano et al. (2020). To the best of our knowledge, this is a novel approach as it combines, in the same study, variables associated with memory and musical processes, where previous works have only shown separately that brain oscillations in the various frequency bands are related to memory processes (Hanslmayr et al., 2019; Herweg et al., 2020).

## 2 Materials and methods

### 2.1 Participants

The sample was taken from the study by López-Cano et al. (2020). It comprised 35 older adults (age range: 61–73 years; Mage = 67.25, SD = 3.47; 22 women). The average number of years of education was 10.48 (SD = 4.68; years range: 5.17 years). Participants were recruited from centres for retired people in Albacete (Spain), where they participate in recreational or cultural activities.

The general exclusion criteria were as follows: (a) more than 4 years of musical practice or academic equivalent (i.e., 1st grade of music school; El Haj et al., 2012); (b) being ill and/or having auditory disorders that would hinder undertaking the tasks; (c) being aged over 73, as we were unable to find songs that reached number one on the charts consulted from before 1959; (d) no active signs of cognitive deficit at the time of assessment, determined by a score of 23 or higher on the Mini-Mental State Examination (Folstein et al., 1975); and (e) presenting depressive symptoms, determined by scores of 11 or higher on the short form of the Patient-Reported Outcomes Measurement Information System (PROMIS-Depression; Cella et al., 2010).

With the available sample, the sensitivity and the required effect size for repeated measures comparisons have been calculated. With values of α err prob. = 0.050, power (1-β err prob) = 0.80 and total sample size (*n* = 35), the parameters obtained are noncentrality parameter *δ* = 2.53, critical t(34) = 1.69, and effect size dz. = 0.43.

### 2.2 Materials

#### 2.2.1 Mini-Mental State Examination (MMSE)

The MMSE is a screening test that quantitatively estimates the existence and severity of cognitive impairment, without providing a diagnosis of any specific nosological entity (Folstein et al., 1975). The maximum score is 30 points, which is obtained by summing the scores on all the items. The cut-off score for cognitive impairment is usually set at 23 points or less.

#### 2.2.2 PROMIS-Depression

This questionnaire consists of four items used to measure the negative affect experienced by an individual in the last 7 days (Cella et al., 2010). Responses range from 1 = never to 5 = always, where the higher the score, the higher is the negative affect. Total scores range from 4 to 20. The cut-off point was set at 11 points. The reliability of this instrument is *α* = 0.96.

#### 2.2.3 Musical cues

The collection of popular songs was compiled by consulting various music databases (Davidalic, 2010; Ramos, 2017, 2020; LOS40, 2018). We included a total of 64 excerpts (32 international hits and 32 Spanish ones) from the “Top 10 music” from the years between 1959 and 1991. Following the procedure of previous works (Janata et al., 2007; Ford et al., 2016), each was 30 s in length. For the music to be highly familiar, the clips were selected taking into account their having reached number 1 on the charts consulted in their year of release. In order to avoid generating, we sought not to repeat groups or singers, although in some years this was not possible due to the lack of diversity in the number ones on the music charts. In addition, we did not select songs that were at the top of the charts in consecutive years. Finally, easily recognizable parts of each song, such as the chorus, were selected. Each participant listened to 10 clips from the first 5 years of their RB and 10 from the 5 years immediately after the end of their RB period. Thus, a person born in 1954 listened to a selection from the RB years (1969–1973) and non-RB years (1985–1989). The range was established following the approximate standard RB period (15–30 years; Berntsen and Rubin, 2002).

#### 2.2.4 Autobiographical memories

To assess the autobiographical memories associated with the musical stimuli, a dichotomous question was asked: memory evoked by the music, yes/no. If the music cue generated a no response, that is, the response either did not correspond to the clip or did not refer to an AM (e.g., “I want to have money), it was classified as a non-memory. Given the exploratory nature of the study, the main aim of the experimental design was to associate the memory with the music to produce a positive effect on memory activation.

#### 2.2.5 Self-assessment manikin (SAM)

This is a self-report questionnaire that assesses emotional response (affective valence, arousal and dominance) (Bradley and Lang, 1994). For this study, we have used the item measuring emotional valence. Thus, the participants rated, on a 9-point Likert-type scale, how pleasant (9) or unpleasant (1) they felt while listening to each music clip. The questionnaire uses graphic figures which represent the different emotional states and is therefore rapid and simple to administer in older people, regardless of participants’ educational level.

### 2.3 Experimental task

**Figure 1:**
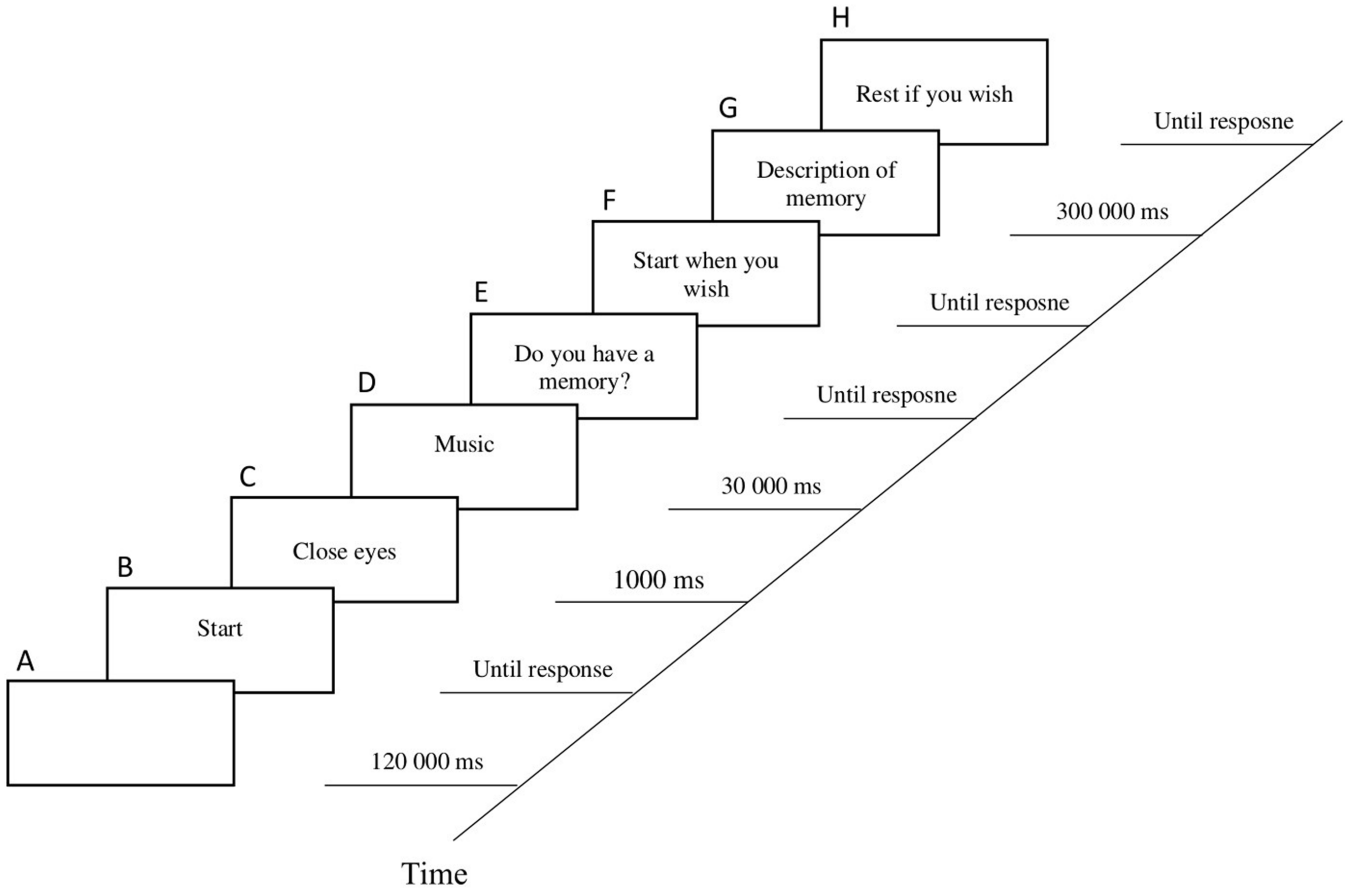
Sequence of the trial in the experimental task. A: Relaxation (2 min). B: Explanation. C: Instruction. D: Song clips (30 s), 20 in total. E: Question on memory with dichotomous response (yes/no). F: Instruction prior to recording the memory. G: Recording of memory. H: Optional pause before the next song clip.

The task was administered individually. The participants sat in front of a desktop computer screen with speakers whose volume could be adjusted. At the beginning of the test, they were asked to keep their eyes closed while each excerpt was played to avoid possible sensory interference. From the beginning of the test until the appearance of the first instruction, there was a two-minute period in which participants were asked to relax and concentrate on their breathing. This was followed by the presentation of the block of 20 clips (10 RB songs and 10 randomly appearing non-RB songs). Each clip lasted 30 s and, following each clip, first, participants completed SAM scale and, second, by means of a dichotomous response question (yes/no), they were asked to indicate whether the music clip generated a memory. In the case of a positive response (i.e., memory generated), when the participant was ready, a process began in which they were recorded recounting the memory. They were asked to provide details, and information on the date and place where it occurred; a time limit of 5 min was set (El Haj et al., 2012). If the participant wished, after retelling the event, they were permitted to rest before starting the next clip (see the trial sequence in Figure 1). The mean duration of the experimental task was 35.4 min (SD = 0.13; range = 24 min–52.8 min).

### 2.4 EEG recording

**Figure 2:**
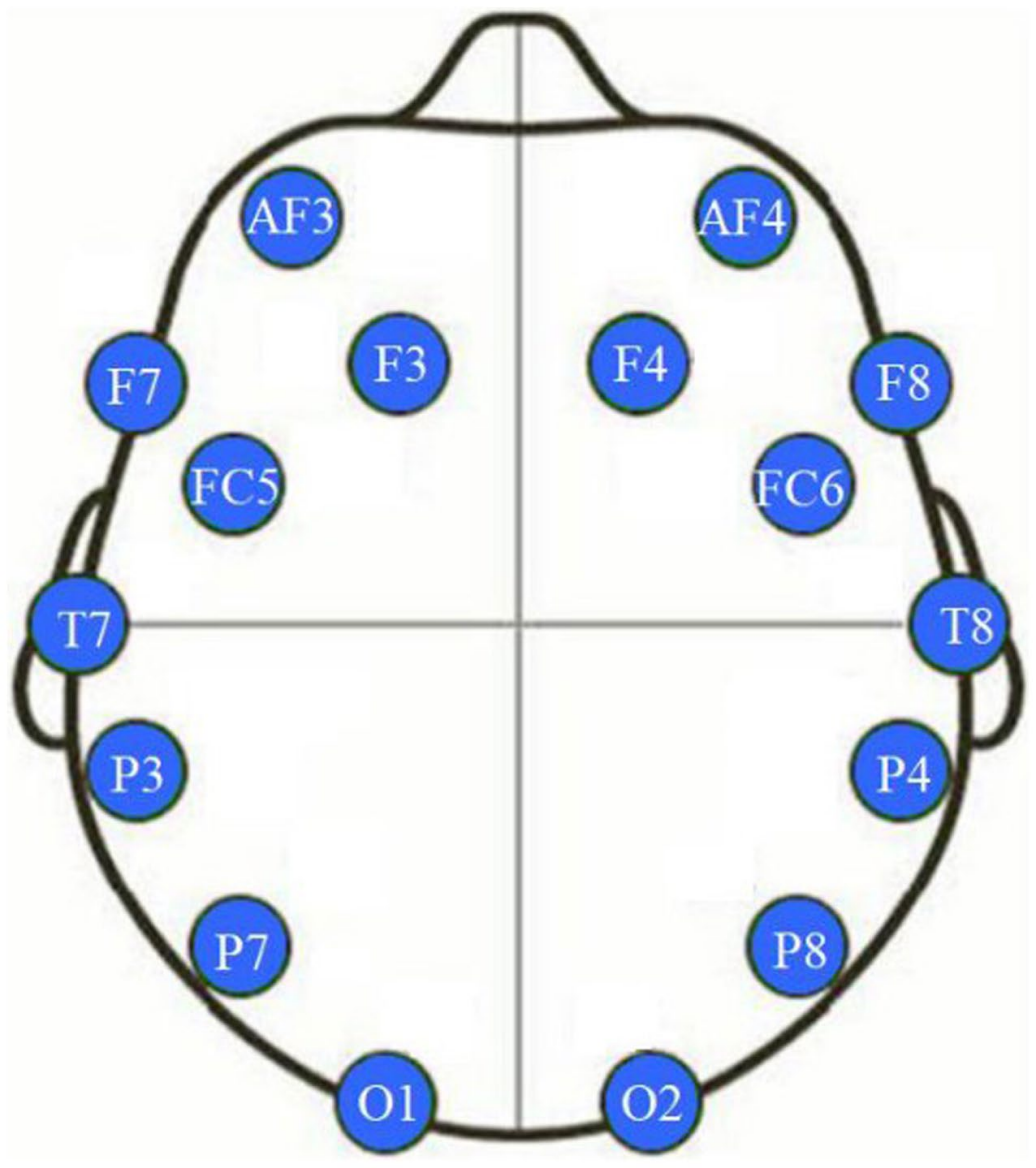
Location of electrodes in the international 10–20 EEG system.

To record the EEG signal, the Emotiv Epoc+ device was used (EPOC+ User Manual, 2019). This device is a brain-computer interface (BCI) with 14 channels and two reference electrodes (P3 and P4), located according to the coordinates of the International 10–20 system (see Figure 2). The sampling frequency of the BCI was 128 Hz. These devices have shown good results in distinguishing dimensional emotional states (Sánchez-Reolid et al., 2022), as well as in the differential response to different musical patterns (Fernández-Soto et al., 2018).

**Figure 3:**
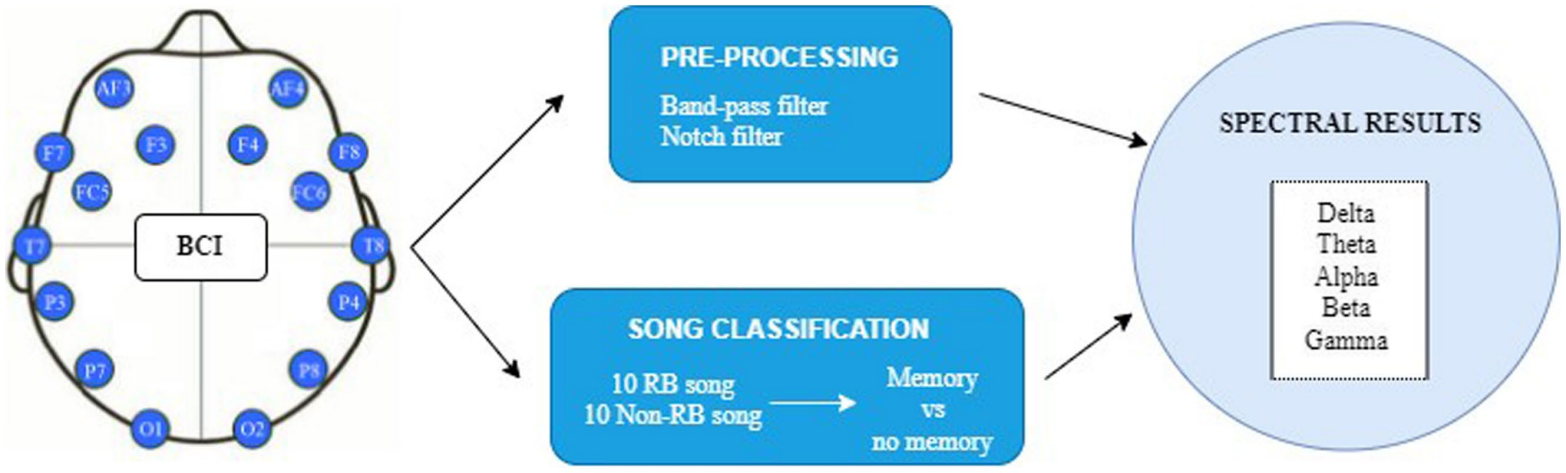
Schematic overview of EEG recording process.

The data analysis was primarily conducted in four stages (see Figure 3 for a schematic overview): (a) Segmentation of the signals to obtain the fragment of the signal corresponding to the generation of the memory, i.e., the signal generated while the participants listened to each of the clips. For this purpose, the 30 s corresponding to the signal generated during the listening of each of the 20 clips were selected for the 35 participants, thus having for each participant 10 fragments of RB songs and 10 of non-RB songs. Additionally, all signal records were classified according to whether the corresponding song generated an autobiographical memory or not. The segmentation was performed using Matlab R2020b software (Moler et al., 1982; Ojeda, 2014); (b) Pre-processing of the signal to eliminate possible artifacts. These artifacts were removed using independent component analysis (ICA; Koelstra et al., 2012; Saeid, 2013) for EEGLAB (Delorme and Makeig, 2004). The ICA method is widely used to determine the part of the signal coming from brain activity and the exogenous part, coming mainly from muscle movements and eye blinking (Li and Principe, 2006; Iversen and Makeig, 2019); (c) Signal Processing using the Matlab application Fully Automated Statistical Thresholding for EEG artifact Rejection (FASTER), for processing the signal fragments of interest (Nolan et al., 2010). In this case, a forward or backward filter was used to eliminate the baseline from each EEG channel, i.e., a linear phase finite impulse response filter was applied. Next, a bandpass filter with cut-off frequencies of 3 Hz and 45 Hz was applied to maintain the frequency bands of interest in the EEG spectrum. Hence, by applying a higher cut-off frequency of 45 Hz, the power line interference component (50 Hz) was also completely removed; and (d) Spectral Analysis, in which the characteristic frequencies of the EEG spectrum were examined: theta (4–8 Hz), alpha (8–12 Hz), beta (13–30 Hz) and gamma (40–45 Hz) (Saby and Marshall, 2012).

Welch’s power spectral density (PSD) was calculated to estimate the power of a signal in the different frequency bands. This procedure uses periodogram spectrum estimates, which are the result of converting a signal from the time domain to the frequency domain. First, each time-domain signal was divided into overlapping sub-sequences through a window (their size controls the bias-variance trade-off of the resulting power spectral density), and the periodogram of each subsequence was then averaged (Proakis and Manolakis, 2007).

### 2.5 Procedure

With the aim of avoiding possible effects of fatigue and boredom, the study was conducted in two individual sessions, separated by approximately a week. In the first session, we collected sociodemographic data, self-reported information on health and auditory status and musical experience. The MMSE and PROMIS Depression scale were also administered. In the second session, we conducted the experimental task, which was designed using the E-Prime 3.0 software. The total duration of the procedure (both sessions) was approximately 90 min.

All the procedures implemented complied with the ethical standards of the Helsinki Declaration of 1975, as revised in 2000. All the participants were appropriately informed of the aims of the study and voluntarily agreed to participate, giving their signed informed consent. The protocol was approved by the Clinical Research Ethics Committee of the Castilla-La Mancha Health Service (agreement number 06/2016).

### 2.6 Data analysis

To determine whether there were statistically significant differences in the activation of the frequency bands depending on whether the song generated an autobiographical memory or not, or whether the song was from the RB period or not, one-factor ANOVAs were conducted separately for each recording electrode. Finally, Bayesian correlations were also calculated to determine any possible relationship between power spectral bands and music clips as an autobiographical memory cue.

## 3 Results

### 3.1 Memory vs. no memory when listening to the songs

**Figure 4:**
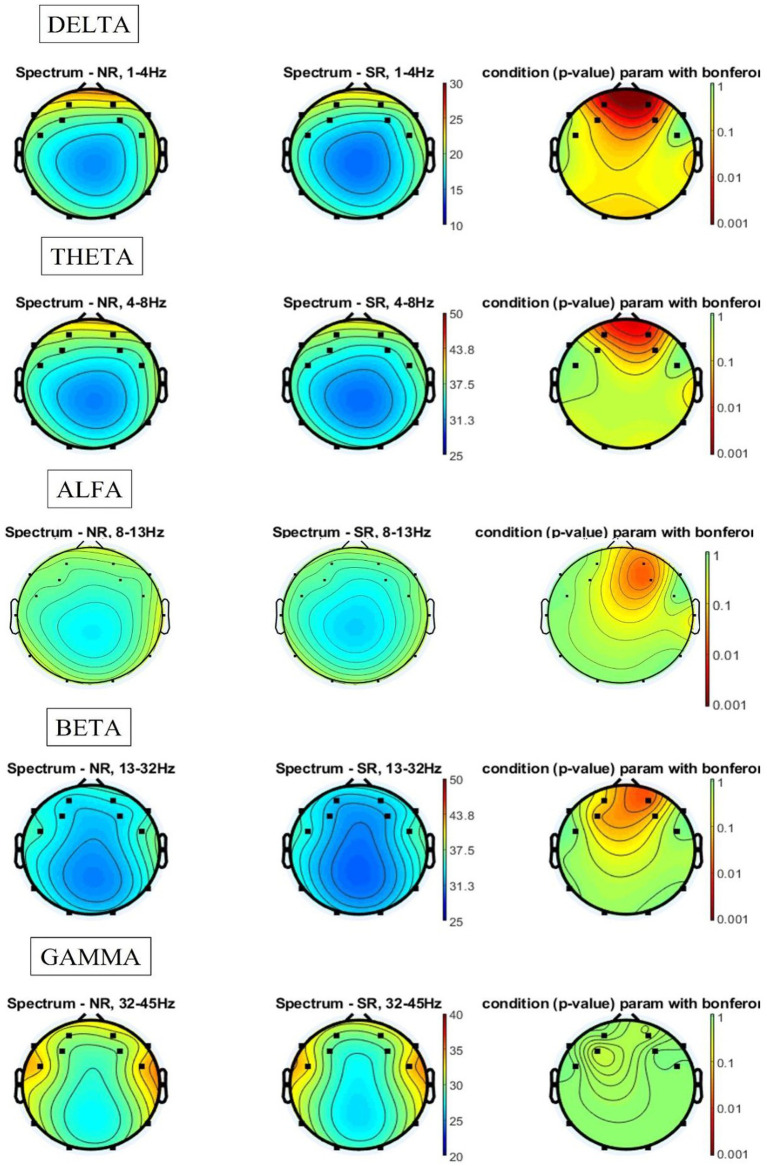
Spectral results for the memory vs. no memory comparison.

Once the experimental task was completed, all signal records corresponding to the 20 music clips per participant were classified according to whether the music clip had and autobiographical memory or not. Out of a total of 700 records, 397 were classified as having generated and autobiographical memory (56.7%). The analysis of the frequency bands of these trials (see Figure 4) showed that, in the case of the delta band, both for the signals collected during the generation of a memory (Mem) and for those obtained while no memory was generated (NoMem), the temporal and frontal areas were activated. When comparing both signals, statistically significant differences were found in the frontal region (electrode AF4), with this area presenting greater activation in the NoMem condition than in the Mem condition.

As regards the theta band, both signals (Mem and NoMem) showed high activation in the frontal region and, to a lesser extent, in the temporal region, although no statistically significant differences were found between the two memory conditions. In the alpha band, frontal activation decreased in both Mem and NoMem, with no differences in activation being found either. In relation to the beta band, both conditions showed a greater decrease in frontal activity and an increase in activation in the temporal regions. Although the difference in activation between Mem and NoMem was mainly located in the area corresponding to electrode AF4, it was not statistically significant. Finally, in the gamma band, a slight increase in frontal region activation was observed for both conditions and, particularly, a greater level of activation in the temporal regions compared to the previous bands, although no differences were found between the two memory conditions (see PSD values for each condition in Supplementary Tables 1, 2).

### 3.2 RB songs vs. non-RB songs

**Figure 5:**
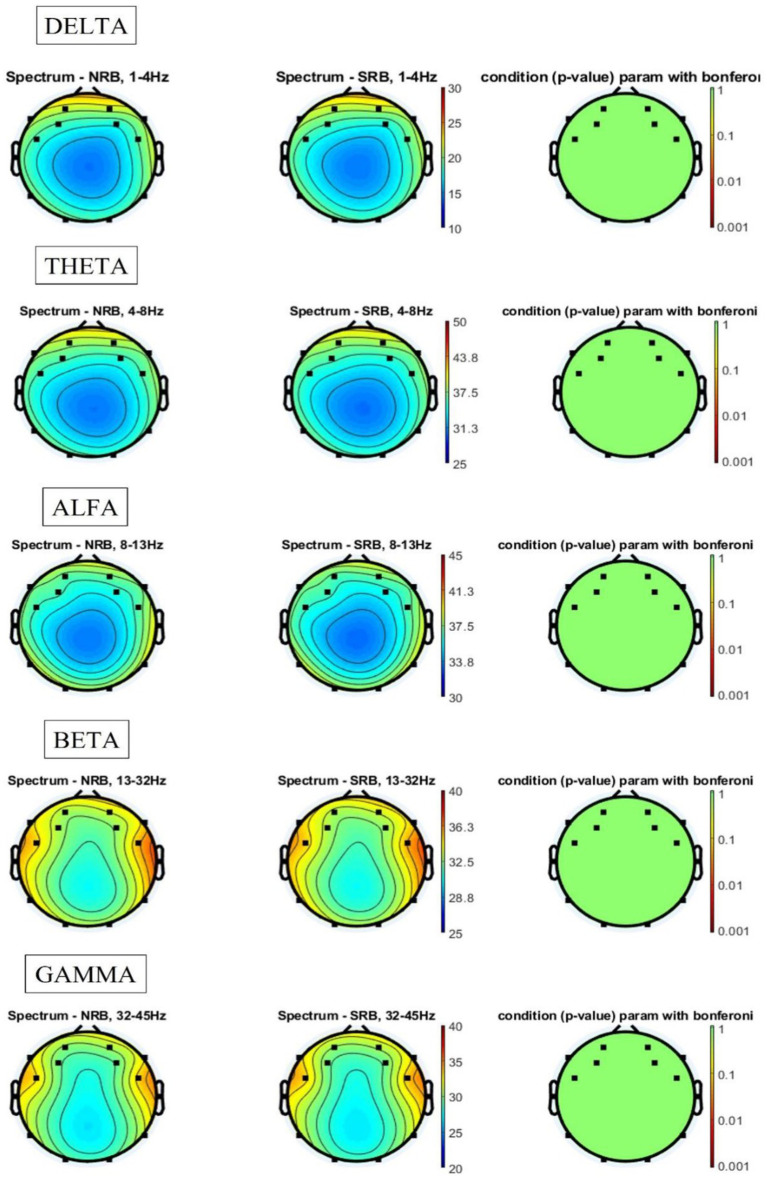
Spectral results for reminiscence bump songs vs. non-reminiscence bump songs.

The comparison of the activation of the frequency bands depending on whether the song was from the RB (350 records: 10 music clips per participant) or non-RB period (350 records: 10 music clips per participant) revealed no statistically significant differences in any of the cases. Figure 5 shows the spectral results of these analyses. In relation to the delta and theta bands, activation was centered in the frontal area, while in the alpha band, we observed the maintenance of frontal activation and the onset of low-level activation in the temporal regions. In the beta band, the activation of both regions (frontal and temporal) increased substantially, especially in the right temporal region. Finally, in the gamma band, the high activation of the temporal regions was maintained, although this was slightly shifted toward the frontal region (see PSD values for each condition in Supplementary Tables 3, 4).

### 3.3 Associations between spectral power bands and music clips

**Figure 6:**
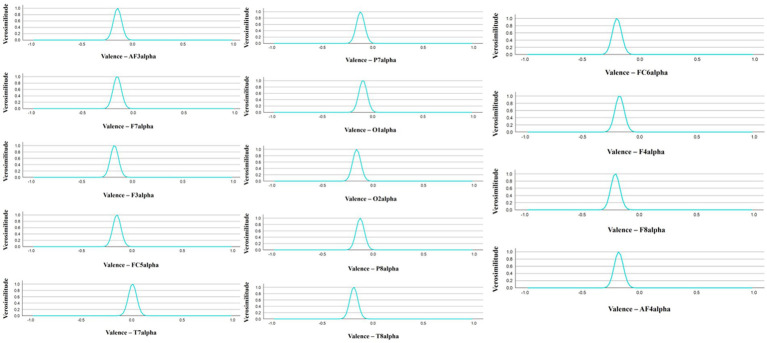
Alpha power band association with music clips emotional valence.

Bayesian correlations between spectral power bands and music clips as an autobiographical memory cue were also analyzed. No significant correlations were found in relation to the presence or absence of song-induced autobiographical recall or in relation to belonging or not to the RB period. Most notable was the negative correlation of alpha spectral power with the emotional valence of the music clips (see Figure 6): there was a decrease in the alpha band associated with a higher emotional valence caused by the songs and the memories associated with them.

## 4 Discussion

The aim of this work was to analyze, in a sample of older adults, the neural processes associated with autobiographical memory induction using popular songs. The main objectives were thus, on the one hand, to determine whether there are differences in the activation of the frequency bands depending on whether or not the music participants listen to is able to generate an associated autobiographical memory, and, on the other, to assess whether the activation of the bands of frequency differs according to whether or not the song is from the RB period.

In relation to the first objective, our results showed higher activation in both conditions (Mem-NoMem) of low frequency bands (delta and theta) in the temporal and frontal areas compared to parietal and occipital regions. Scientific evidence suggests that temporal areas are related to memory and auditory processing tasks (Kroupi et al., 2011), and that frontal regions is associated with emotional responses (Aftanas et al., 2004) and episodic memory control processes (Old and Naveh-Benjamin, 2008). In this line, previous studies have observed an increase in the delta and theta bands in the temporal and prefrontal areas during autobiographical memory tasks (Fuentemilla et al., 2014; Imperatori et al., 2014). As mentioned by Knyazev (2012), the increase of delta power may be associated with attention, particularly the detection of motivationally salient cues in the environment. Nevertheless, the delta band activation was significantly higher in the frontal region (electrode AF4) in the NoMem condition compared to the Mem condition. According to Old and Naveh-Benjamin (2008), older adults have greater difficulties recalling context (who, where, when and how) than content (what). This finding is consistent with the associative deficit hypothesis (Naveh-Benjamin, 2000), which suggests that the difficulty in binding together different pieces of information is a major component of age-related cognitive decline in the episodic memory domain. It is possible that the higher activation in the NoMem trials of the frontal region compared to the Mem trials reflects the use of a generative autobiographical retrieval, which would lead to an increased demand on cognitive resources with the purpose of remembering the appropriate context to generate the memory. Additionally, as mentioned by Cabeza and Dennis (2013), greater activity is not always associated with better cognitive performance. According to these authors, it is unclear whether increased activity in older adults reflects compensation, non-selective recruitment (inability to recruit specialized brain regions) or just a confound in task difficulty.

On the other hand, compared to what was observed with the low frequency bands, the high frequency bands (alpha, beta, and gamma) showed a lower activation of the frontal zones. Specifically, in relation to the alpha band, it showed slight activation of frontal, temporal and occipital areas, finding no differences in this activation between the Mem and NoMem conditions. According to Knyazev et al. (2015), the increase of alpha power in prefrontal regions may be associated with integration of cortical areas, which are directly involved in the reconstruction of autobiographical memories, whereas its increase in occipital areas may reflect inhibition of irrelevant areas that may supply an input, which is apt to interrupt the ongoing mental process (Klimesch et al., 2007). Regarding beta and gamma bands, these showed a slight activation of prefrontal and temporal areas, although gamma band is highly activated in the temporal area. According to Mu and Han (2010), self-relevant items may generate event-related synchronization in beta and gamma-band activity. In this line, Herrmann et al. (2004) propose that high-frequency neural oscillations mediate the comparison of memory contents with stimulus-related information and the utilization of signals derived from this comparison.

As regards the second objective related to the activation of the frequency bands when listening to an RB or non-RB song, we found no statistically significant differences in the activation patterns according to the type of song. Our results showed that both categories of music clips generated an activation of the frontocentral region for low wave frequencies (delta and theta). Moreover, as the wave frequency increased (alpha, beta and gamma), frontal activation increased and the temporal regions, mainly in the right area, were activated. Previous studies have shown that familiar and emotionally stimulating music may have specific neural pathways that activate psychological mechanisms related to episodic memory (Rapadas, 2015), and thus it would be the delta band that shows greater brain activation in response to MEAMs related to the familiarity of the song and episodic memory.

Meanwhile, Sammler et al. (2007) also proposed that music enjoyment was positively correlated with the power spectral density in the theta band over the prefrontal cortex (4–7 Hz). Other studies have found that listening to positively valenced music generated left frontal activation, whereas negatively valenced music activated the right frontal region (Schmidt and Trainor, 2001; Altenmüller, 2002; Sammler et al., 2007). Recently, Nemati et al. (2019) highlighted that listening to pleasant music was associated with relatively higher oscillatory activity in the theta band over the frontocentral region, which could be explained by the fact that that listening to pleasant music demands greater attentional effort, leading to decreased memory performance. That is, according to Nemati et al., the gradual development of low-frequency oscillations in the frontal region may be partly due to the gradual recruitment of higher levels of attention in response to pleasurable music. Additionally, Daly et al. (2014) reported that the pleasantness or unpleasantness generated by the songs was also related to frequencies in the range of 18–22 Hz (within the beta band), finding significant differences between the groups of high pleasantness and high unpleasantness, with a lower right-hemisphere-oriented asymmetry for clips generating higher pleasantness.

Finally, our results also showed that the positive emotional valence was negatively associated with alpha power band. Scientific literature suggests that mere exposure to emotion-laden stimuli has been related to alpha-band desynchronization (Popov et al., 2012). According to Arana et al. (2021), this effect could be explained by an automatic attentional mechanism triggered by emotionally salient stimuli, which would be reflected in the modulation of alpha oscillations. In their study, results showed that self-reported ratings of emotional valence correlated with alpha band: the more negative the valence, the lower the alpha band power. It has to be noted that the age range of their participants was 19–28 years and, as mentioned by Thomas and Hasher (2006), young adults tend to show a stronger negativity bias. Nevertheless, according to the theory of socioemotional selectivity, as people grow older, they typically attend to, and remember, more positive information than negative information, and tend to experience fewer negative emotions (Mather and Carstensen, 2005). Thus, regarding cognitive processing, this theory posits that, with age, individuals focus more on emotionally relevant information and reallocate their processing resources toward the positive aspects of situations rather than the negative ones (Löckenhoff and Carstensen, 2004). This positivity bias could explain why, in our results, positive emotional valence is associated with a decrease in alpha band activation.

### 4.1 Limitations of the study

This study is not without limitations. Firstly, the BCI device used, despite having been shown to be effective in detecting variations in the emotional response generated by music, has some drawbacks compared to standard EEG equipment. Future research might use portable EEG devices with a larger number of channels and a higher signal sampling rate. The second limitation is related to the difficulty in controlling each person’s individual variables, which could impact the results; for example, the subjectivity the music generates in emotions and autobiographical memories (López-Cano et al., 2020). Thirdly, despite the large selection of songs, as they are taken from different periods depending on the age of the participant, the musical patterns and rhythms do not coincide, which could bias the results. Future studies might analyze the EEG response invoked by songs selected by the participants themselves. Finally, for future research, as regards the retrieval of autobiographical memories, it would be useful to analyze the differences between men and women (in line with the work of Manns et al., 2018), and also between young and old people, since older people show lower beta activity than do young people (Laera et al., 2021).

## 5 Conclusion

In conclusion, brain responses are formed by the combined presence of processes that can be related to musical perception, memory retrieval and affect (Hadjidimitriou and Hadjileontiadis, 2012). The results show that, in general, there are scant differences in the activation of brain frequency bands depending on whether the music clip can induce an autobiographical memory or whether it belongs to the RB or not. The only difference found suggests that older adults show increased activation of low frequency waves in the frontal region, indicating they are making a greater cognitive effort in generative autobiographical retrieval. However, this effort seems to be insufficient to evoke a memory. Future studies should focus not only on comparing brain activation, but also on assessing differences in functional brain connectivity. In this regard, Cabeza and Dennis (2013) suggest that, with aging, frontal cortex connectivity might increase to compensate for the neural decline associated with aging. It is possible that the differences in brain function when evoking the memories elicited by music clips are at the level of neural connectivity rather than activation.

## Data availability statement

The datasets presented in this study can be found in online repositories. The names of the repository/repositories and accession number(s) can be found at: OSF repository (https://doi.org/10.17605/OSF.IO/ATUCR).

## Ethics statement

The studies involving humans were approved by Clinical Research Ethics Committee of the Castilla-La Mancha Health Service (agreement number 06/2016). The studies were conducted in accordance with the local legislation and institutional requirements. The participants provided their written informed consent to participate in this study.

## Author contributions

MM-S: Data curation, Formal analysis, Investigation, Writing – original draft, Writing – review & editing. LR: Formal analysis, Funding acquisition, Methodology, Supervision, Writing – original draft, Writing – review & editing. ML-C: Investigation, Writing – original draft, Writing – review & editing. MN: Methodology, Writing – original draft, Writing – review & editing. BN: Methodology, Writing – original draft, Writing – review & editing. JML: Conceptualization, Formal analysis, Funding acquisition, Supervision, Writing – original draft, Writing – review & editing.
