# Supplementary material for: Effect of popular songs from the reminiscence bump as autobiographical memory cues in aging: a preliminary study using EEG
Source: Front Neurosci. 2024 Jan 9;17:1300751. doi: 10.3389/fnins.2023.1300751 (PMC10803499; doi:10.3389/fnins.2023.1300751)
Supplement: Supplementary file 1 [file Table_1.docx]

**Supplementary Material**

**Table 1**

*Spectral power values in* *W/Hz* *for the memory generated group*

| Frequency | AF3 | F7 | F3 | FC5 | T7 | P7 | O1 | O2 | P8 | T8 | FC6 | F4 | F8 | AF4 |
| --- | --- | --- | --- | --- | --- | --- | --- | --- | --- | --- | --- | --- | --- | --- |
| Delta | 0.17 | 0.21 | 0.08 | 0.09 | 0.16 | 0.08 | 0.08 | 0.04 | 0.06 | 0.08 | 0.08 | 0.04 | 0.16 | 0.13 |
| Theta | 14.95 | 15.39 | 9.31 | 7.72 | 17.80 | 10.03 | 8.19 | 5.16 | 6.99 | 8.08 | 8.24 | 5.14 | 11.45 | 10.56 |
| Alpha | 12.33 | 11.68 | 15.85 | 8.90 | 21.42 | 17.70 | 22.32 | 20.72 | 20.90 | 17.43 | 10.56 | 13.03 | 10.45 | 10.40 |
| Beta | 17.29 | 31.87 | 23.68 | 37.02 | 44.32 | 21.62 | 17.37 | 15.80 | 23.55 | 46.62 | 59.32 | 22.23 | 39.88 | 17.22 |
| Gamma | 7.53 | 20.36 | 10.13 | 22.47 | 19.25 | 8.44 | 4.54 | 4.25 | 9.45 | 22.52 | 37.17 | 10.89 | 26.10 | 7.52 |

*Note*. Spectral power values obtained from the 30-second duration of the music clips that have generated an autobiographical memory.

AF3, F7, F3, FC5, T7, P7, O1, O2, P8, T8, FC6, F4, F8 and AF4 refer to the names of the 14 BCI channels, positioned according to the international 10/20 system.

**Table 2**

*Spectral power values in W/Hz for the no memory generated group*

| Tipo | AF3 | F7 | F3 | FC5 | T7 | P7 | O1 | O2 | P8 | T8 | FC6 | F4 | F8 | AF4 |
| --- | --- | --- | --- | --- | --- | --- | --- | --- | --- | --- | --- | --- | --- | --- |
| Delta | 0.16 | 0.23 | 0.11 | 0.06 | 0.06 | 0.07 | 0.08 | 0.06 | 0.07 | 0.12 | 0.06 | 0.05 | 0.19 | 0.15 |
| Theta | 11.79 | 13.21 | 9.76 | 6.09 | 6.92 | 7.05 | 9.06 | 5.59 | 6.76 | 9.74 | 7.03 | 7.13 | 14.83 | 11.56 |
| Alfa | 13.05 | 13.05 | 19.09 | 10.28 | 16.25 | 18.54 | 24.59 | 21.35 | 21.95 | 24.67 | 12.90 | 19.25 | 12.07 | 13.13 |
| Beta | 21.02 | 28.92 | 30.73 | 46.48 | 29.36 | 23.96 | 22.93 | 19.30 | 24.62 | 75.93 | 71.75 | 34.82 | 34.08 | 27.63 |
| Gamma | 9.09 | 16.01 | 14.08 | 23.23 | 14.46 | 7.86 | 5.98 | 4.12 | 8.33 | 25.32 | 42.02 | 14.55 | 18.30 | 10.91 |

*Note*. Spectral power values obtained from the 30-second duration of the music clips that have not generated an autobiographical memory.

AF3, F7, F3, FC5, T7, P7, O1, O2, P8, T8, FC6, F4, F8 and AF4 refer to the names of the 14 BCI channels, positioned according to the international 10/20 system.

**Table 3**

*Spectral power values in W/Hz for the reminiscence bump period songs condition.*

| Frequency | AF3 | F7 | F3 | FC5 | T7 | P7 | O1 | O2 | P8 | T8 | FC6 | F4 | F8 | AF4 |
| --- | --- | --- | --- | --- | --- | --- | --- | --- | --- | --- | --- | --- | --- | --- |
| Delta | 0.13 | 0.17 | 0.08 | 0.05 | 0.10 | 0.08 | 0.08 | 0.05 | 0.06 | 0.08 | 0.07 | 0.04 | 0.19 | 0.13 |
| Theta | 11.77 | 12.61 | 8.58 | 5.45 | 10.60 | 10.23 | 8.03 | 5.39 | 7.23 | 8.17 | 7.83 | 5.15 | 12.26 | 11.31 |
| Alpha | 12.88 | 11.44 | 16.96 | 8.52 | 13.83 | 18.82 | 23.85 | 21.39 | 22.13 | 18.73 | 11.11 | 14.80 | 10.68 | 11.66 |
| Beta | 17.56 | 27.95 | 23.74 | 31.04 | 27.93 | 21.48 | 17.34 | 15.86 | 23.93 | 39.91 | 53.90 | 21.16 | 33.36 | 18.28 |
| Gamma | 7.77 | 18.02 | 10.09 | 20.42 | 15.74 | 8.01 | 4.04 | 3.74 | 9.29 | 20.99 | 34.07 | 9.98 | 21.31 | 7.92 |

*Note*. Spectral power values obtained from the 30-second duration of the music corresponding to RB songs.

AF3, F7, F3, FC5, T7, P7, O1, O2, P8, T8, FC6, F4, F8 and AF4 refer to the names of the 14 BCI channels, positioned according to the international 10/20 system.

**Table 4**

*Spectral power values in W/Hz for the non-reminiscence bump period songs condition.*

| Type | AF3 | F7 | F3 | FC5 | T7 | P7 | O1 | O2 | P8 | T8 | FC6 | F4 | F8 | AF4 |
| --- | --- | --- | --- | --- | --- | --- | --- | --- | --- | --- | --- | --- | --- | --- |
| Delta | 0.19 | 0.26 | 0.11 | 0.11 | 0.14 | 0.13 | 0.08 | 0.05 | 0.06 | 0.08 | 0.07 | 0.05 | 0.16 | 0.14 |
| Theta | 15.32 | 16.18 | 11.04 | 8.27 | 16.06 | 10.84 | 8.98 | 5.11 | 6.37 | 7.96 | 7.63 | 6.45 | 13.30 | 10.48 |
| Alpha | 11.55 | 12.11 | 17.29 | 9.71 | 24.29 | 17.83 | 21.79 | 20.20 | 19.98 | 19.19 | 11.64 | 15.93 | 10.97 | 10.78 |
| Beta | 15.62 | 29.31 | 25.69 | 36.59 | 45.96 | 20.43 | 18.54 | 15.70 | 20.16 | 41.50 | 59.50 | 23.92 | 36.19 | 18.30 |
| Gamma | 7.08 | 18.52 | 10.54 | 22.42 | 18.29 | 7.77 | 5.35 | 3.93 | 7.93 | 22.00 | 36.70 | 11.28 | 23.96 | 8.20 |

*Note*. Spectral power values obtained from the 30-second duration of the music corresponding to non-RB songs.

AF3, F7, F3, FC5, T7, P7, O1, O2, P8, T8, FC6, F4, F8 and AF4 refer to the names of the 14 BCI channels, positioned according to the international 10/20 system.
